# Electrophysiological analysis of mammalian cells expressing hERG using automated 384-well-patch-clamp

**DOI:** 10.1186/s40360-015-0042-9

**Published:** 2015-12-16

**Authors:** Yuji Haraguchi, Atsushi Ohtsuki, Takayuki Oka, Tatsuya Shimizu

**Affiliations:** Institute of Advanced Biomedical Engineering and Science, TWIns, Tokyo Women’s Medical University, 8-1 Kawada-cho, Shinjuku-ku, Tokyo 162-8666 Japan; Nanion Technologies GmbH, Gabrielenstr.9, 80636 Munich, Germany

**Keywords:** Electrophysiology, hERG channel, High-throughput screening, Automated patch-clamp

## Abstract

**Background:**

An in vitro electrophysiological assay system, which can assess compound effects and thus show cardiotoxicity including arrhythmia risks of test drugs, is an essential method in the field of drug development and toxicology.

**Methods:**

In this study, high-throughput electrophysiological recordings of human embryonic kidney (HEK 293) cells and Chinese hamster ovary (CHO) cells stably expressing human ether-a-go-go related gene (hERG) were performed utilizing an automated 384-well-patch-clamp system, which records up to 384 cells simultaneously. hERG channel inhibition, which is closely related to a drug-induced QT prolongation and is increasing the risk of sudden cardiac death, was investigated in the high-throughput screening patch-clamp system.

**Results:**

In the automated patch-clamp measurements performed here, K_v_ currents were investigated with high efficiency. Various hERG channel blockers showed concentration-dependent inhibition, the 50 % inhibitory concentrations (IC_50_) of those blockers were in good agreement with previous reports.

**Conclusions:**

The high-throughput patch-clamp system has a high potential in the field of pharmacology, toxicology, and cardiac physiology, and will contribute to the acceleration of pharmaceutical drug development and drug safety testing.

## Background

At present although the cost of pharmaceutical drug development has been progressing, new pharmaceutical drugs finally approved and launched into the market is decreasing steadily. In 2001, while 30 % of pharmaceutical drugs, which were tested clinically, were abandoned because of the lack of efficacy, 30 % of others were also abandoned because of safety concerns such as cardiotoxicities including ion channel inhibition [1, 2]. Ion channels are major targets of pharmaceutical drugs, it is shown that more than 13 % of clinically used drugs act primarily on ion channel proteins, these drugs are estimated to be worth more than $12 billion worldwide [3]. Therefore, the development of an in vitro electrophysiological assay system, which can detect the efficacy of candidate drugs or cardiotoxicity including arrhythmia risks is strongly demanded in the field of pharmacological development and drug safety testing. An assay system using cells expressing human ion channels has a powerful potential when it comes to reducing the amount of animal experiments. In addition, utilizing cells expressing human ion channels is expected to be an accurate assessment, because there might be different reactivity against drugs between human and animal ion channels. For example, the heartbeat of a mouse is around 600 beats per minute, which is tenfold faster than that of human beings, thus, the duration of the action potential is much shorter and ion channels have different properties [4]. Furthermore, the usage of mammalian cells expressing human ion channel genes is more suitable than Xenopus oocytes expressing the genes, which may be less sensitive to drug inhibition [5–7].

A patch-clamp system allows for investigation of the electrophysiological function of ion channels, and was first described by Neher and Sakmann who were awarded the Nobel Prize in Medicine in 1991 [8, 9]. While the technology is an essential method in the field of pharmacology, toxicology, and cardiac physiology, a conventional patch-clamp setup is generally thought to be demanding and needs high levels of manual dexterity, knowledge and dedication of the experimenter. An automated patch-clamp system on the other hand is easy to use compared to conventional patch-clamping [10–12].

In this study a high-throughput electrophysiological screening of human embryonic kidney (HEK 293) cells and Chinese hamster ovary (CHO) cells stably expressing human ether-a-go-go related gene (hERG) was performed utilizing an automated 384-well-patch-clamp system. hERG channel inhibition of various blockers was analyzed, and the 50 % inhibitory concentrations (IC_50_) were compared to literature values.

## Methods

### Cell culture and cell preparation for patch-clamp analysis

In this study, HEK 293-hERG cells (Merck Millipore, Billerica, MA, USA), which are HEK 293 cells stably expressing hERG, and CHO-hERG cells (Merck Millipore), which are CHO cells stably expressing hERG, were used. The HEK 293 cells were cultured in an equal volume mixture of Dulbecco’s modified Eagle’s medium (DMEM) and Nutrient Mixture F-12 (Invitrogen Life Technologies, CA, USA) supplemented with 10 % fetal bovine serum (FBS) (Invitrogen Life Technologies) and 1 % penicillin/streptomycin (Invitrogen Life Technologies), and the CHO cells were cultured in Ham’s F12 medium (Invitrogen Life Technologies) supplemented with 10 % FBS and 1 % penicillin/streptomycin in a humidified 5 % CO_2_ atmosphere at 37 °C. For patch-clamp experiments, cultured cells on a polystyrene culture dish (Sumitomo Bakelite, Tokyo, Japan) were detached by Accutase (Innovative Cell Technologies, Inc., CA, USA) at room temperature for several minutes. A viable single cell suspension was obtained by resuspension in patch clamp solution with mild pipetting to avoid cell damage.

### Patch-clamp analysis

**Fig. 1:**
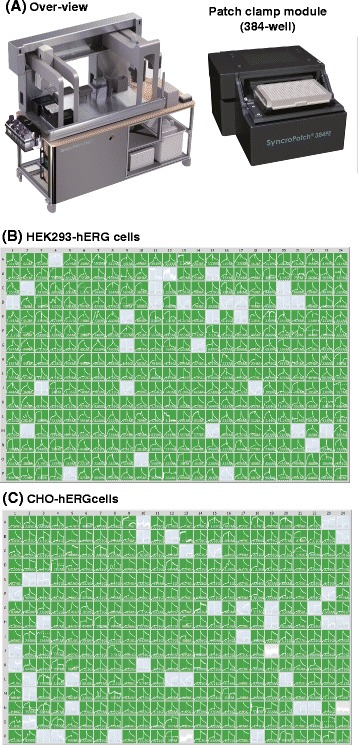
Automated 384-well-patch-clamp system. **a** Left and right photographs show the over-view and the patch-clamp module having 384-wells of the system, respectively. Those two photographs in (**a**) are under the copyright of Nanion Technologies GmbH, and have been used with permission from the company. The system could measure membrane currents up to 384 cells simultaneously (**b** and **c**). The numbers in (**b**) and (**c**) are the values of their seal resistances. For example, the value, “567 M”, of 1A column in (**b**) is “567 MΩ”, and that, “3.43 G”, of 1B column in (**c**) is “3.43 GΩ”. Green or gray panels show complete experiments or incomplete experiments, respectively

Patch-clamp measurements were performed by an automated multi-well planar patch-clamp system the SyncroPatch 384 Patch Engine (PE) (Nanion Technologies, Munich, Germany) (Fig. 1a). The SyncroPatch 384 PE was a patch-clamp module that was integrated into a liquid handling robot owning a 384 pipetting head, Biomek FX (Beckman Coulter, Brea, CA, USA). The system was controlled by a dedicated software, PatchControl 384 (Nanion Technologies). In this device, 384 cells were measured simultaneously. Patch-clamp recordings were performed at room temperature using an intracellular solution [50 mM KCl, 10 mM NaCl, 60 mM KF, 20 mM EGTA, 10 mM HEPES (pH: 7.2) with 25 μM Escin for perforated patch (HEK 293-hERG cells) or without Escin for whole cell recording (CHO-hERG cells)], and a standard bath solution [140 mM NaCl, 4 mM KCl, 1 mM MgCl_2_, 2 mM CaCl_2_, 5 mM D-glucose, 10 mM HEPES (pH: 7.4)]. Suspended single cells and drugs were freshly prepared and applied into teflon reservoirs (Nanion Technologies). In the automated patch-clamp system, the following procedures were performed automatically. The suspended single cells were aspirated from the reservoir, pipetted into a planar 384-well patch-clamp chip, and entrapped in the holes of the wells by an automatically applied vacuum. Seal generation, the establishment of the perforated or standard whole cell mode and also the electrophysiological recordings were controlled by PatchControl 384. The application of drugs into each well and the washout were also performed automatically by the liquid handling robot. Concentration response curves and IC_50_ values were calculated automatically by another dedicated software, DataControl 384 (Nanion Technologies). K_v_ currents were elicited using a voltage step from a holding potential (-80 mV) to +20 mV for 1 s followed by a 1 s step to -50 mV.

### hERG channel blockers

In this study, five hERG channel blockers [astemizole (Sigma-Aldrich, St. Louis, MO, USA), cisapride monohydrate (Sigma-Aldrich), E-4031 hydrochloride (Wako pure chemical, Tokyo, Japan), quinidine (Sigma-Aldrich), and terfenadine (Sigma-Aldrich)] were used. Three concentrations of each drug were applied to the same cell cumulatively and sequentially. Three hundred eighty-four wells could be served with solution at the same time due to the availability of the 384 pipetting head of the system.

## Results

### Measurement of K_v_ currents in an automated 384-well-patch-clamp system

A patch-clamp investigation of HEK 293 cells or CHO cells expressing hERG was performed in an automated way by using a 384-well-patch-clamp system. For the analysis, 10 μL of suspended cells (cell density: approximately 1 × 10^6^ cells/mL) were applied to each well. A complete experiment took approximately 15–25 min. K_v_ currents were efficiently detected in the voltage-clamp mode of the high-throughput system (Fig. 1b, c).

### High-throughput assay on hERG inhibitors

**Fig. 2:**
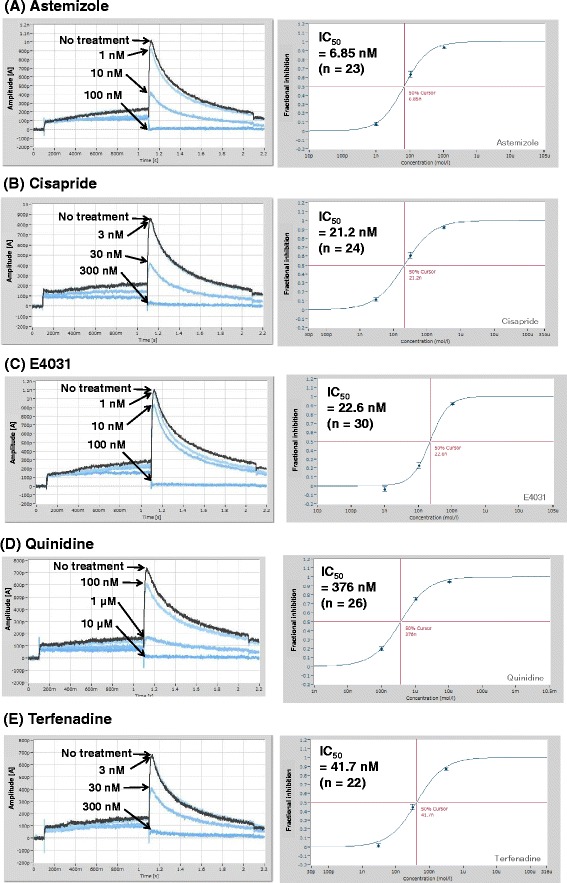
Inhibition of hERG currents by various hERG channel blockers. hERG currents on HEK 293 cells expressed hERG gene were detected by an automated 384-well-patch-clamp system. hERG currents were inhibited concentration-dependently by five hERG channel blockers, astemizole (**a**), cisapride (**b**), E-4031 (**c**), quinidine (**d**), and terfenadine (**e**). Left and right panels show the representative recordings of hERG current inhibition and the inhibitory curves of hERG currents, respectively. The 50 % inhibitory concentrations (IC_50_) were analyzed and shown in the left upper sides of the right panels. The n numbers were total numbers of cells at all concentrations of each drug. Three hundred eighty-four points were divided into six, and five hERG channel blockers and no blocker were added. The error bars in the right panels show means ± SD and IC_50_ values show means

The high-throughput system was used to investigate a number of hERG channel inhibitors. The assay was performed using five hERG channel blockers, which were two antiarrhythmic drugs [E-4031 (Class III) and quinidine (Class Ia)] and three noncardiovascular drugs [astemizole (antihistamine), cisapride (gastrokinetic), and terfenadine (antihistamine)]. hERG currents were inhibited by those drugs in a concentration dependent way (Fig. 2a-e). The IC_50_ of those drugs were summarized in Table 1. Those values were in good agreement with previous reports (Table 1), showing the feasibility of the automated high-throughput patch-clamp system when performing a hERG screening.

**Table 1:** IC50 of hERG channel blockers

Reagents	IC_50_	
	Data in this study	Previous data (References)
Astemizole	6.85 nM (n = 23)	0.9 nM (2), 26 nM (4)
Cisapride	21.2 nM (n = 24)	44 nM (1), 6.9 nM (4), 23-27 nM (5)
E-4031	22.6 nM (n = 30)	18.1 nM (4), 12-17 nM (5)
Quinidine	376 nM (n = 26)	410 nM (3), 820-1,070 nM (5), 750 nM (6)
Terfenadine	41.7 nM (n = 22)	56 nM (1), 6.6-8.4 nM (5), < 52 nM (7)

References for Table 1.

1. Lacerda AE, *et al.*: *Eur Heart J Supplements* 2001, **3:**K23-K30.

2. Zhou Z, *et al.*: *J Cardiovasc Electrophysiol* 1999, **10:**836-843.

3. Paul AA, *et al.*: *Br J Pharmacol* 2002, **136:**717-729.

4. Chiu PJ, *et al*.: *J Pharmacol Sci* 2004, **95:**311-319.

5. Kirsch GE, *et al*.: *J Pharmacol Toxicol Methods* 2004, **50:**93-101.

6. Wolpert C, *et al*.: *J Cardiovasc Electrophysiol* 2005, **16:**54-58.

7. Katchman AN, *et al*.: *J Pharmacol Exp Ther* 2006, **316:**1098-1106.

## Discussion

This study showed data of an automated patch-clamp system, which records ion channel currents of up to 384 cells simultaneously. The system could detect hERG channel inhibition in a high-throughput format using HEK 293 cells overexpressing hERG channels. The hERG channel is characterized as a voltage-gated inwardly rectifying potassium channel [13, 14], and plays a key role in cardiac pathology because the gene links to long QT syndrome, which is a hereditary disease causes lethal ventricular arrhythmias [15–17]. Importantly, the channel inhibition causes a drug-induced QT prolongation and is increasing the risk of sudden cardiac death [5, 15–21]. Of drugs recently removed from the market in the United States, one of the most common causes has been QT prolongation-related cardiotoxicity [22]. Therefore, an optimal evaluation system of hERG channel blockers is important for detecting the cardiotoxicity of candidate drugs. hERG channel screening of candidate drugs at an early stage in the drug development process is accelerating the whole drug discovery procedure. This study is proposing a high-throughput screening system for investigating hERG channel inhibition using an automated multi-well-patch-clamp technology. The patch-clamp method allows for the simultaneous assessment of ion channel inhibition activity of e.g. up to 48 or 128 kinds of candidate drugs, in the case of n = 8 or 3, respectively.

It is commonly thought that the usage of human cardiomyocytes is also important in the field of pharmacological development and drug safety testing [2, 4, 23]. Human induced pluripotent stem cells (hiPSC) can efficiently differentiate into cardiomyocytes in vitro [24]. We developed a suspension culture system, which can produce large numbers of hiPSC-derived cardiomyocytes [25]. hiPSC-derived cardiomyocytes have been applied for cardiac regenerative medicine and the transplantation of an enormous number of the cells will contribute to positive clinical therapeutic effects [24]. At the same time those cardiomyocytes will be also an optimal cell source for the high-throughput investigation of ion channel inhibition and thus the detection of cardiotoxicity of drugs. Our previous report showed that hiPSC-derived cardiomyocytes expressed various cardiac cell-related genes, including hyperpolarization activated cyclic nucleotide-gated potassium channel 4 (HCN4), myosin light chain-2a (MLC-2a), MLC-2v, and Iroquoishomeobox 4 (IRX4) [26]. HCN4 is expressed in cardiac pacemaker cells [27]. MLC-2a is a marker of atrial myocytes, and MLC-2v and IRX4 are those of ventricular myocytes [28]. Thus, the data suggest that hiPSC-derived differentiated cells contained various types of cardiomyocytes including pacemaker cells, atrial and ventricular myocytes. Currently, we are performing the patch-clamp analysis of hiPSC-derived differentiated cardiomyocytes, the amount of cells being expanded abundantly by the suspension culture system, using the automated 384-well-patch-clamp system. An upgrade of the here utilized 384-well-patch-clamp system to not only having the capability of performing voltage-clamp, but also current-clamp recordings is momentarily under development. With this system the effect of candidate drugs on the duration of the action potentials will be investigated, which could be translated into e.g. a prolongation of QT intervals. Additionally, the system will allow high-throughput recordings of cardiac subtypes including pacemaker cells, atrial myocytes, ventricular myocytes, and also will allow for investigating the maturation status of hiPSC-derived cardiomyocytes. Those data will contribute to the field of cardiac electrophysiology and cardiac regenerative medicine as well as pharmaceutical development.

## Conclusion

This study shows data from a hERG screening assay in an automated high-throughput patch-clamp system. We are confident that the method will have great impact in the field of pharmacology, toxicology, and cardiac electrophysiology, also in the light of the CIPA (Comprehensive In Vitro Pro-Arrhythmia Assay) proposal that aims to define a new, integrated preclinical in vitro/*in silico* paradigm in which the potential proarrhythmic risk of a new drug would be assessed using not only hERG patch clamp investigations, but multiple ion channel investigations (e.g. Na_v_1.5 and Ca_v_1.2). Thus, the system will contribute to the acceleration of pharmaceutical drug-development and drug-safety testing.
